# Relationship between emergency nurses’ professional competencies and the Nursing care product*

**DOI:** 10.1590/1518-8345.6585.3939

**Published:** 2023-06-02

**Authors:** Jucinei Araújo de Jesus, Alexandre Pazetto Balsanelli

**Affiliations:** 1 Universidade Federal de São Paulo, Escola Paulista de Enfermagem, São Paulo, SP, Brasil.

**Keywords:** Employee Performance Appraisal, Process Assessment, Health Care, Professional Competence, Emergency Nursing, Nursing Administration Research, Professional Practice, Evaluación de Desempeño Profesional, Evaluación de Procesos en Atención de Salud, Competencia Profesional, Enfermería de Urgencia, Investigación en Administración de Enfermería, Práctica Profesional, Avaliação de Desempenho Profissional, Avaliação de Processos em Cuidados de Saúde, Competência Profissional, Enfermagem em Emergência, Pesquisa em Administração de Enfermagem, Prática Profissional

## Abstract

**Objective:**

to relate urgency and emergency nurses’ professional competencies with the Nursing care product.

**Method:**

a cross-sectional study conducted in the urgency and emergency units of two public hospitals. The participants were 91 nurses, 3 Nursing residents, 4 coordinators and 1 manager. Two validated instruments were used: 1) Competence Scale of Actions of Nurses in Emergencies and 2) Nursing Care Product Evaluation. Factors and domains were used, respectively. Descriptive statistics were applied, as well as Cronbach’s alpha, Wilcoxon and Spearman’s correlation tests (p<0.05).

**Results:**

in the professional competencies, higher values were verified for self-evaluation (p<0.001). In all 1,410 Nursing care product assessments, there was predominance of the “Good” score (n=1,034 - 73.33%). The “Nursing staffing” domain was related to the “Professional practice” (r=0.52719), “Relationships at work” (r=0.54319), “Positive challenge” (r=0.51199), “Targeted action” (r=0.43229), “Constructive behavior” (r=0.25601) and “Adaptation to change” (r=0.22095) factors; the “Care monitoring and transfer” domain, with “Professional practice” (r=0.47244), “Relationships at work” (r=0.46993), “Positive challenge” (r=0.41660) and “Adaptation to change” (r=0.31905) and the “Meeting care needs” domain, with “Professional practice” (r=0.32933), “Relationships at work” (r=0.31168), “Positive challenge” (r=0.29845) and “Adaptation to change” (r=0.28817).

**Conclusion:**

there is a relationship between professional competencies and the Nursing care product domains.

## Abstract (es)

**Objetivo:**

relacionar las competencias profesionales de los enfermeros de urgencia y emergencia con el producto del cuidado de enfermería.

**Método:**

estudio transversal, realizado en las unidades de urgencia y emergencia de dos hospitales públicos. Participaron 91 enfermeros, 3 residentes de enfermería, 4 coordinadores y 1 gestor. Se utilizaron dos instrumentos validados: 1) Escala de Competencia de las Acciones de los Enfermeros en Emergencias y 2) Evaluación del Producto del Cuidado de Enfermería. Se utilizaron factores y dominios, respectivamente. Se aplicaron estadística descriptiva, alfa de Cronbach, pruebas de Wilcoxon y correlación de Spearman (p<0,05).

**Resultados:**

para las competencias profesionales se registraron valores más altos en la autoevaluación (p<0,001). En las 1.410 evaluaciones del producto del cuidado de enfermería, prevaleció el puntaje “bueno” (n=1034 - 73,33%). El dominio “dimensionamiento del personal de enfermería” se relacionó con los factores “práctica profesional” r=0,52719, “relaciones en el trabajo” r=0,54319, “desafío positivo” r=0,51199, “acción dirigida” r=0,43229, “conducta constructiva” r=0,25601 y “adaptación al cambio” r=0,22095; el dominio “seguimiento y transferencia del cuidado”, con “práctica profesional” r=0,47244, “relaciones en el trabajo” r=0,46993, “desafío positivo” r=0,41660 y “adaptación al cambio” r=0,31905 y el dominio “satisfacción de las necesidades asistenciales”, con “práctica profesional” r=0,32933, “relaciones en el trabajo” r=0,31168, “desafío positivo” r=0,29845 y “adaptación al cambio” r=0,28817.

**Conclusión:**

hay relación entre las competencias profesionales y los dominios del producto del cuidado de enfermería.

## Abstract (pt)

**Objetivo:**

relacionar as competências profissionais do enfermeiro em urgência e emergência com o produto do cuidar em enfermagem.

**Método:**

estudo transversal, realizado nas unidades de urgência e emergência de dois hospitais públicos. Participaram 91 enfermeiros, 3 residentes em enfermagem, 4 coordenadores e 1 gerente. Utilizaram-se dois instrumentos validados: 1) Escala de Competências das Ações dos Enfermeiros em Emergências e 2) Avaliação do Produto do Cuidar em Enfermagem. Utilizaram-se fatores e domínios, respectivamente. Aplicaram-se estatística descritiva, alfa de Cronbach, testes Wilcoxon e correlação de Spearman (p<0,05).

**Resultados:**

em competências profissionais, verificaram-se maiores valores para autoavaliação (p<0,001). Nas 1.410 avaliações do produto do cuidar em enfermagem, predominou o escore “bom” (n=1034 - 73,33%). O domínio “dimensionamento de pessoal de enfermagem” relacionou-se com os fatores “prática profissional” r=0,52719, “relações no trabalho” r=0,54319, “desafio positivo” r=0,51199, “ação direcionada” r=0,43229, “conduta construtiva” r=0,25601 e “adaptação à mudança” r=0,22095; o domínio “acompanhamento e transferência do cuidado”, com “prática profissional” r=0,47244, “relações no trabalho” r=0,46993, “desafio positivo” r=0,41660 e “adaptação à mudança” r=0,31905 e o domínio “atendimento das necessidades assistenciais”, com “prática profissional” r=0,32933, “relações no trabalho” r=0,31168, “desafio positivo” r=0,29845 e “adaptação à mudança” r=0,28817.

**Conclusão:**

existe relação entre as competências profissionais e os domínios do produto do cuidar em enfermagem.

**Highlights:**

**(1)** The professional competencies are related to the Nursing care product.

**(2)** Staffing in APROCENF was related to six CSANE factors.

**(3)** Care transfer in APROCENF was related to four CSANE factors.

**(4)** Staffing and care transfer require competencies.

## Introduction

Nurses operates in various segments in the intra- and extra-hospital spheres, preparing, organizing, coordinating and implementing care actions, whose purpose is to enable rehabilitation of the patients as well as their reintegration into family and social life^(1)^. These professionals provide care as a science and as an art, grounding their actions on technical-scientific knowledge and raising the bar of the profession’s assumptions^(2)^.

In their centrality, urgency and emergency units have certain complexity that characterizes the Brazilian health system which, considering the service as one of the gateways to the Unified Health System (*Sistema* Único *de Saúde*, SUS), requires professionals who are skilled and dynamic in clinical reasoning as well as in decision-making, in order to perform effectively and efficiently in the problems presented by the patients, who seek care for their health conditions^(3)^.

In this sense, nurses act as care managers in urgency and emergency units, through the specific competencies required, such as leadership, decision-making, clinical reasoning and effective communication, among others, for operationalization in the unit’s care process. In their role as care managers, nurses plan a range of actions that will be transformed into assistance, centralizing these activities into specific demands, where the patients will be the main consumers, that is, directed care is consumed as soon as produced^(4-5)^.

Therefore, it is evident that, in order to manage care, specific competencies are required, skills that, supported on knowledge, will enable the patient’s rehabilitation and integration process. In this case, nurses are care providers to the extent that they develop and employ competencies to turn it into a consumable. Such competencies were described in a matrix^(6)^ which identified them as crucial for nurses’ performance in urgency and emergency units, gathering and consolidating the dimensions of the care provided in these units from a theoretical framework.

After designing and implementing the care measures, these professionals work with the multiprofessional team in order to share the required work demand, by means of the treatment and rehabilitation possibilities and, with this, conferring even more robustness to the action plan which will be demanded from standardization of the language spoken by the entire multiprofessional team, characterizing the patients’ real health needs^(7)^.

That said, it becomes imperative to qualify care and verify how much the implemented actions are exerting an impact on meeting the patients’ needs, delivering what is indispensable through the competencies that guide this entire process. Once planning is concluded, it is expected to deliver a product^(5)^ worthy of the efforts undertaken in its elaboration and that then be used by the patient. New studies should be carried out with a view to further exploring the nurses’ competencies in urgencies and emergencies, strengthening their skills with a view to the care product that they should deliver at the end of the work period.

Based on the aforementioned proposals, the specific question of the current study was as follows: Is there any relationship between urgency and emergency nurses’ competencies and the Nursing care product? A systematic review identified nonexistence of such relationship^(8)^. Consequently, in order to answer this question, the objective defined was to relate urgency and emergency nurses’ professional competencies with the Nursing care product.

## Method

### Study design

This is a cross-sectional and correlational study^(9)^, with its design based on the STrengthening the Reporting of OBservational studies in Epidemiology (STROBE) guideline^(10)^.

### Data collection locus

The study was carried out in the urgency and emergency units of two public hospitals, one of them a university hospital and the other one a secondary-level hospital with tertiary-level characteristics, both references of the Urgency and Emergencies Network (*Rede de Urgências e Emergências*, RUE) care line. The hospitals are respectively located in the South and Southwest regions of the municipality of São Paulo-SP, Brazil and they were identified as Hospital A and Hospital B. Both services were randomly chosen to provide for the inclusion of more participants.

### Period

The study was developed between June and December 2020.

### Sample

The convenience sample consisted of 91 nurses, 3 Nursing residents, 4 coordinators and 1 manager.

### Selection criteria

The professionals included were those working in urgency and emergency units with a minimum employment contract of three months, as well as Nursing residents attending second year of the same area. Nurses working in the sector only as a stopover were excluded, as well as Nursing residents from other areas and those who were on vacation and on leave.

In the emergency sector of Hospital A, 53 nurses were eligible and were invited to take part in the study. However, one of them withdrew his agreement to participate during the research. Consequently, 52 nurses from Hospital A were included (96.29% response rate). Of these, 49 professionals were clinical nurses and three were Nursing residents in the urgency and emergency area, working in the morning, afternoon and night shifts, even and odd and taking turns in emergency, risk classification, medical clinic observation and surgical clinic observation, corridor and medication. Three nurses responsible for the sector (1 manager and 2 Nursing coordinators) were invited to carry out the hetero-evaluation of the professional competencies.

In turn, in Hospital B, 42 individuals (95.45% response rate) were invited and participated in the research, with two exclusions (1 due to medical leave and 1 for being on vacation). The sector had 36 nurses on duty, distributed in the morning, afternoon and night shifts, characterized as even and odd and eight day laborers, providing assistance in risk classification, emergency room, suture, shock room, urgent care, adult ward, children’s first aid and ward, rear and psychiatric observation. A total of 42 nurses were eligible for the study. Both of the nurses in charge of sector’s team (Nursing coordinators) were also invited to take part in the research, meeting hetero-evaluation.

Therefore, at Hospital A, the participants were 55 nurses (52 for self-evaluation and 3 for hetero-evaluation) and, at Hospital B, 44 nurses (42 for self-evaluation and 2 for hetero-evaluation), totaling 99 participants.

### Instruments used for data collection

The Competence Scale of Actions of Nurses in Emergencies (*Escala de Competências das Ações dos Enfermeiros em Emergências -* ECAEE)^(6)^ and the Nursing Care Product Assessment Scale (*Avaliação do Produto do Cuidar em Enfermagem*, APROCENF)^(5)^ were used, both made in Brazil and validated for their use on the Brazilian population.

The ECAEE^(6)^ is made up of 78 items representing nurses’ actions in emergencies, divided into seven factors: Factor 1 - “Professional practice” (33 items); Factor 2 - “Relationships at work” (19 items); Factor 3 - “Positive challenge” (10 items); Factor 4 - “Targeted action” (7 items); Factor 5 - “Constructive behavior” (2 items); Factor 6 - “Professional excellence” (4 items) and Factor 7 - “Adaptation to change” (3 items). For each item mentioned, the nurses answered considering their self-evaluation according to a Likert scale from 1 to 5 (Extremely Competent, Very competent, Competent, Little competent, and Not competent). In the hetero-evaluation, the Nursing coordinators and managers answered the instruments with the same items applied to the nurses; however, in their assessment, the nurses’ performance was considered.

The APROCENF scale^(5)^ consists of eight domains: Nursing care planning; Resources needed to provide assistance; Nursing staffing; Educational actions and staff development; Care monitoring and transfer; Multidisciplinary interaction and action; Care provided to the patient and/or family member and Meeting the care needs. Each item is followed by a progressive scale that varied from one to four, where the higher the score, the better the Nursing care product. The values individually obtained in each item are added up and generate a classification according to the following ranges: 9-12 points (Poor); 13-21 (Fair); 22-30 (Good) and 31-32 (Excellent).

### Study variables

The variables used were age, gender, marital status, year of graduation in Nursing, *lato sensu* and *stricto sensu* graduate studies, improvement, certification, residency, Bachelor’s degree, emergency courses, other participations in courses and events, scientific activities, allocation sector, work shift and double employment contract, as well as the final scores and the domains and factors from the data collection instruments were used.

### Data collection

A pre-test were performed, in which 10 nurses (Hospital A=5; Hospital B=5) were randomly selected in order to verify possible difficulties answering the instruments. After raising awareness about the study, its importance and feasibility for the Nursing practice, ECAEE^(6)^ was handed in and, after verifying there were no difficulties filling it out, the participants received the APROCENF scale^(5)^, not finding any difficulties. After this phase, data collection was initiated at Hospital A, where awareness was again raised with an explanation of the research objectives and clarification of how to answer the instrument ECAEE. Concomitantly, the managers also received the same guidelines and instrument to conduct the nurses’ hetero-evaluation. Subsequently, the research was applied in Hospital B, with the same guidelines and explanations.

Once this stage was concluded, application of the APROCENF scale was initiated. The nurses were instructed in relation to its filling-in, always at the end of the work shift and on alternating days. All 94 nurses were instructed to answer the APROCENF scale fifteen times, that is, after fifteen shifts, to obtain more assessments portraying the sector’s reality as well as based on the validation study corresponding to the APROCENF scale^(5)^, generating a total of 1,410 assessments of the care product. It is worth noting that the nurses took turns in all the urgency and emergency sectors. The 5 nurses performing coordination and management roles did not answer the APROCENF scale.

### Data treatment and analysis

To describe the sample profile, tables of variables were prepared with absolute (n) and percentage (%) frequency values, as well as descriptive statistics with mean values, standard deviation, minimum and maximum values, median and quartiles. In order to assess internal consistency of the scales, Cronbach’s alpha coefficient was used, with values from 0.70 considered satisfactory^(11)^. For data treatment, the IBM Statistical Package for the Social Sciences (SPSS) software was used, version 22 in Portuguese, in addition to requesting the help of a professional statistician.

Spearman’s correlation coefficient^(9)^ was used to assess the relationship between the variables corresponding to the total scores and to the factors and domains from the data collection instruments. Wilcoxon’s test for related samples was employed to compare the self-evaluation and hetero-evaluation scores. The Intraclass Correlation Coefficient (ICC) was used to assess inter- and intra-evaluator reliability. It is worth noting that the relationship between the ECAEE factors and the domains from the APROCENF scale considered the mean of the 15 assessments of the APROCENF scale, performed by each nurse.

The significance level adopted for the statistical tests was 5%, that is, p<0.05.

### Ethical aspects

The study was developed according to the following stages: 1-) Authorization of the main authors of the data collection instruments; 2-) Authorization of the hospitals that served as study field; 3-) Registration on *Plataforma Brasil* and opinion by the Research Ethics Committee (*Comitê de* Ética *em Pesquisa*, CEP) of the Federal University of São Paulo (*Universidade Federal de São Paulo*, UNIFESP) (No. 3,317,669).

The nurses and residents working in the emergency sectors were invited to take part in the research. They accepted by signing the Free and Informed Consent Form. The data collection instruments were handed in to these professionals, printed and in envelopes, to be later collected on an agreed upon day.

## Results

The hospitals where the study was developed were identified as A and B. The total number of professionals who answered the survey was n=99 nurses, distributed as follows: Hospital A, n=55; and Hospital B, n=44.

The nurses’ overall descriptive analysis was performed considering both hospitals. Thus, regarding age, n=52 (55.32%) respondents were between 30 and 39 years old, with predominance of females, n=70 (74.47%); n=49 (52.13%) declared themselves to be single, to the detriment of n=32 (34.04%) who stated being married. Table1 indicates the characterization of the sample.

**Table 1 -:** Descriptive analysis of the ECAEE^*^ categorical variables corresponding to the urgency and emergency nurses (n=94). São Paulo, SP, Brazil, 2020

Variable		n	%
Year of graduation in Nursing	1991-2001	4	4.2
	2002-2012	45	48.9
	2013-2018	45	47.9
*Lato sensu* graduate studies	Yes	73	77.7
	No	21	22.3
*Stricto sensu* graduate studies	Yes	7	7.5
	No	87	92.6
Improvement	Yes	2	2.1
	No	92	97.9
Certification	Yes	16	17.0
	No	78	83.0
Residency	Yes	7	7.8
	No	87	92.6
Bachelor’s degree	Yes	-	-
	No	94	100.0
Emergency coursesattended (in the last 2 years)	Yes	39	40.9
	No	55	59.1
Other participations	Yes	22	23.4
	No	72	76.6
Scientific activities	Yes	23	24.5
	No	71	75.5

*ECAEE = *Escala de Competências das Ações dos Enfermeiros em Emergências* (The Competence Scale of Actions of Nurses in Emergencies)

In relation to the sector, n=88 (93.62%) were allocated to Emergency Room for Adults (ERA), whereas n=6 (6.38%) carried out their activities exclusively in the ER medical clinic, with their work schedules were distributed among the morning (7 am-1 pm) with n=16** **(17.02%), afternoon (1 pm-7 pm) with n=19 (20.21%) and night (7 pm-7 am) periods with n=38 (40.43%) as well as day shift workers (7 am-7 pm) with n=21 (22.34%).

In relation to working as a nurse in another institution, n=63 (67.02%) stated not having any other employment contract. Of n=31 (32.98%) that did have it, the majority worked from 7 am to 7 pm (n=18, 58.06%) and allocated to the ERA units (n=11, 35.48%).

Regarding *lato sensu* graduate studies, of the 73 nurses with specialist degrees, n=65 (67.12%) of the respondents had a specialization in the Nursing area and, of these, n=37 (56.92%) were specialized in urgency and emergency. In turn, regarding *stricto sensu* graduate studies, five participants had finished their MSc courses and two were PhDs in the Nursing and Health Sciences areas. Two nurses had completed improvement courses in Home Health and Nursing Service Management; n=16 (17.02%) said that they had completed qualification in passing a peripherally inserted central catheter (PICC); n=7 (7.45%) completed their residency, with n=4 in the urgency and emergency field, n=1 in Intensive Care, n=1 in Nephrology and n=1 in Internal Medicine and Surgery. Regarding the update courses in the last two years, n=38 (40.86%) reported courses such as Basic Life Support (BLS), Advanced Cardiac Life Support (ACLS), Advanced Trauma Life Support (ATLS FOR NURSE), Advanced Trauma Care For Nurse (ATCN FOR NURSE), Prehospital Trauma Life Support (PHTLS), Advanced Trauma Support Operations (*Manobras Avançadas de Suporte ao Trauma*- MAST), Pediatric Advanced Life Support (PALS) and the patient classification protocol. In relation to other types of participation, n=22 (23.40%) answered that they had participated in the organization of events, study groups, commissions, committees and scientific events, in the last two years.

Regarding scientific activities, n=23 (24.47%) answered that they had participated in the elaboration of scientific papers, publication of scientific papers in congress annals, publication of scientific paper abstracts and scientific articles published in journals and distributed between 2003 and 2019.

Table2 below presents the comparisons of the competence level scores between the self- and hetero-evaluations. Given the results, a significant difference was verified between the scores, with higher values for self-evaluation. The ICC (inter-evaluator reliability) between both scores was 0.511, indicating significant agreement (different from zero) between the evaluators (self- and hetero-evaluation).

**Table 2 -:** Comparative evaluation of the urgency and emergency nurses (n=94) between the self- and hetero-evaluation scores from the ECAEE^*^. São Paulo, SP, Brazil, 2020

Variable	n	Mean	SD^†^	Min^‡^	Q1^§^	Median	Q3^║^	Max^¶^	***p***-value**	ICC^††^	(95% CI^‡‡^, ICC^††^)
Total self-evaluation	94	330.97	13.51	300.00	320.00	331.00	339.00	369.00	p=0.010	0.511	(0.342; 0.648) p<0.001
Total hetero-evaluation	94	325.68	23.07	272.00	306.00	335.50	344.00	359.00			
Difference between self- and hetero-evaluation	94	5.29	18.35	-35.00	-10.00	1.50	20.00	40.00			

*ECAEE = *Escala de Competências das Ações dos Enfermeiros em Emergências* (The Competence Scale of Actions of Nurses in Emergencies); ^†^SD = Standard Deviation; ^‡^Min = Minimum; ^§^Q1 = Quartile 1; **^║^** Q3 = Quartile 3; ^¶^Max = Maximum; ^**^p-value = Referring to Wilcoxon’s test for related samples for the comparison between both scores; ^††^ICC = Intraclass Correlation Coefficient to measure the agreement between both scores; ^‡‡^CI = Confidence Interval

Table3 presents the frequency and descriptive statistics of the entire sample for the APROCENF scale (n=94), considering all 15 assessments made by each nurse (n=1.410). The nurses concentrated their care actions taking turns in all the emergency sectors during their respective work shifts. The rating that was most emphasized by the nurses in the item scale corresponded to number three, indicating a “Good” APROCENF score (73.3%).

**Table 3 -:** Descriptive analysis corresponding to the categorical variables from the APROCENF^*^(n=1.410) scale among the urgency and emergency nurses. São Paulo, SP, Brazil, 2020

Items from the APROCENF* scale	Rating	Frequency	Percentage
APROCENF 1 Nursing care planning	1	65	4.61
	2	313	22.20
	3	955	67.73
	4	77	5.46
APROCENF 2 Necessary resources to provide care	1	98	6.95
	2	296	20.99
	3	919	65.18
	4	97	6.88
APROCENF 3 Nursing staffing	1	30	2.13
	2	453	32.13
	3	874	61.99
	4	53	3.76
APROCENF 4 Educational actions and professional development	1	99	7.02
	2	240	17.02
	3	976	69.22
	4	95	6.74
APROCENF 5 Care monitoring and transfer	1	33	2.34
	2	412	29.22
	3	914	64.82
	4	51	3.62
APROCENF 6 Multidisciplinary interaction and performance	1	83	5.89
	2	265	18.79
	3	983	69.72
	4	79	5.60
APROCENF 7 Care provided to the patient and/or family member	1	110	7.80
	2	226	16.03
	3	1,009	71.56
	4	65	4.61
APROCENF 8 Meeting the care needs	1	101	7.16
	2	227	16.10
	3	993	70.43
	4	89	6.31
APROCENF Total	-	-	-
Fair	-	375	26.60
Good	-	1,034	73.33
Excellent	-	1	0.07

^*^APROCENF = *Avaliação do Produto do Cuidar em Enfermagem* (Nursing Care Product Assessment Scale)

Good internal consistency was obtained (alpha>0.70)^(10)^ in the following ECAEE factors: Factor 1 (*0.790*) and Factor 2 (*0.720*); this difference was significant in the hetero-evaluation carried out by the Nursing service managers. In all 15 assessments performed by the nurses, Cronbach’s alpha was 0.501.

Tables4and5 below present the correlations between the domains from the APROCENF scale (considering the 15 assessments made by each nurse) and the ECAEE items, for the self- and hetero-evaluation. The significant correlations are highlighted in the table, emphasizing the variables where significant differences were obtained.

**Table 4 -:** Correlations between the ECAEE* variables (self-evaluation) and the domains from the APROCENF^†^ (n=1.410), scale corresponding to the urgency and emergency nurses. São Paulo, SP, Brazil, 2020

Factors	Nursing care planning	Necessary resources to provide care	Nursing staffing	Educational actions and professional development	Care monitoring and transfer	Multidisciplinary interaction and performance	Care provided to the patient and/or family member	Meeting the care needs	APROCENF^†^ total
Self- F1^‡^ Professional practice	r^§^=0.1488	0.0946	0.4490	0.1551	0.3381	0.2337	0.1172	0.2747	0.4673
	p=0.1522	0.3644	<0.0001	0.1353	0.0009	0.0234	0.2605	0.0074	<0.0001
Self- F2^║^ Relationships at work	0.1361	0.0628	0.3570	-0.0127	0.1332	0.1141	0.1139	0.0569	0.2510
	0.1907	0.5473	0.0004	0.9030	0.2003	0.2732	0.2742	0.5856	0.0147
Self- F3^¶^ Positive challenge	0.0198	-0.1366	0.1213	0.0904	0.0656	0.0100	-0.0120	0.1675	0.1021
	0.8494	0.1891	0.2438	0.3859	0.5296	0.9234	0.9085	0.1064	0.3272
Self- F4** Targeted action	0.0417	-0.1473	0.0696	0.0135	0.1060	-0.0435	0.0364	-0.0294	0.0484
	0.6896	0.1563	0.5049	0.8967	0.3091	0.6771	0.7271	0.7783	0.6431
Self- F5^††^ Constructive behavior	0.1181	0.0760	0.1034	0.0154	0.1034	-0.1472	-0.2132	0.2065	0.0642
	0.2568	0.4665	0.3210	0.8827	0.3209^|^	0.1568	0.0391	0.0457	0.5387
Self- F6^‡‡^ Professional excellence	-0.0772	0.0195	-0.0094	0.0615	0.0549	0.0308	-0.0625	0.0898	0.0267
	0.4596	0.8514	0.9281	0.5560	0.5987	0.7681	0.5495	0.3893	0.7977
Self- F7^§§^ Adaptation to change	-0.0667	0.1208	-0.0005	-0.0288	-0.0614	-0.0194	0.0713	-0.1486	-0.0407
	0.5229	0.2461	0.9958	0.7827	0.5566	0.8523	0.4945	0.1528	0.6964

*ECAEE = *Escala de Competências das Ações dos Enfermeiros em Emergências* (The Competence Scale of Actions of Nurses in Emergencies); ^†^APROCENF = *Avaliação do Produto do Cuidar em Enfermagem* (Nursing Care Product Assessment Scale); ^‡^Self- F1 Professional practice; ^§^r = Spearman’s correlation coefficient; ^║^Self- F2 Relationships at work; ^¶^Self- F3 Positive challenge; ^**^Self- F4 Targeted action; ^††^Self- F5 Constructive behavior; ^‡‡^Self- F6 Professional excellence; ^§§^Self- F7 Adaptation to change

**Table 5 -:** Correlations between the ECAEE^*^ variables (hetero-evaluation) and the domains from the APROCENF^†^ scale (n=1,410) corresponding to the urgency and emergency nurses. São Paulo, SP, Brazil, 2020

Factors	Nursing care planning	Necessary resources to provide care	Nursing staffing	Educational actions and professional development	Care monitoring and transfer	Multidisciplinary interaction and performance	Care provided to the patient and/or family member	Meeting the care needs	APROCENF^†^ total
Hetero- F1^‡^ Professional practice	r^§^=0.2615	0.2316	0.5271	0.1919	0.4724	0.2359	0.1500	0.3293	0.6121
	p=0.0109	0.0247	<0.0001	0.0638	<0.0001	0.0220	0.1488	0.0012	<0.0001
Hetero- F2^║^ Relationships at work	0.1856	0.2503	0.5431	0.2645	0.4699	0.1950	0.1576	0.3116	0.5912
	0.0732	0.0150	<0.0001	0.0100	<0.0001	0.0596	0.1291	0.0022	<0.0001
Hetero- F3^¶^ Positive challenge	0.2663	0.2307	0.5119	0.2561	0.4166	0.2732	0.0679	0.2984	0.5805
	0.0095	0.0253	<0.0001	0.0127	<0.0001	0.0077	0.5150	0.0035	<0.0001
Hetero- F4** Targeted action	0.1841	0.1603	0.4322	0.1776	0.1681	-0.0301	0.1021	0.1831	0.3666
	0.0757	0.1227	<0.0001	0.0867	0.1053	0.7732	0.3273	0.0773	0.0003
Hetero- F5^††^ Constructive behavior	0.1208	0.1910	0.2560	0.0984	0.1891	0.1427	0.0827	0.1879	0.3306
	0.2459	0.0651	0.0128	0.3450	0.0678	0.1700	0.4278	0.0697	0.0011
Hetero- F6^‡‡^ Professional excellence	-0.0278	-0.0938	0.0571	-0.0251	-0.1304	0.0594	0.0865	0.0363	0.0140
	0.7900	0.3684	0.5844	0.8096	0.2101	0.5692	0.4066	0.7281	0.8933
Hetero- F7^§§^ Adaptation to change	0.2102	-0.0048	0.2209	0.1562	0.3190	0.1551	0.1464	0.2881	0.3186
	0.0419	0.9633	0.0324	0.1325	0.0017	0.1354	0.1589	0.0049	0.0017

*ECAEE = *Escala de Competências das Ações dos Enfermeiros em Emergências* (The Competence Scale of Actions of Nurses in Emergencies); ^†^APROCENF = *Avaliação do Produto do Cuidar em Enfermagem* (Nursing Care Product Assessment Scale); ^‡^Hetero- F1 Professional practice; ^§^r = Spearman’s correlation coefficient; ^║^Hetero- F2 Relationships at work; ^¶^Hetero- F3 Positive challenge; **Hetero- F4 Targeted action; ^††^Hetero- F5 Constructive behavior; ^‡‡^Hetero- F6 Professional excellence; ^§§^Hetero- F7 Adaptation to change

## Discussion

Professional competencies are part of the development of the Nursing team. The profession is based on a profile that confers a solid identity to the entire category, detailing what is incumbent to each one. For nurses who have the tasks of organizing, preparing, structuring and implementing the Nursing care service, these competencies are fundamental elements that affect care provision by these professionals in each shift, prioritizing the patients’ care needs^(12)^. However, this provision takes place from technical-scientific thinking based on diverse knowledge that guides the profession^(13)^.

The population of this study was predominantly female, not escaping the scope of Brazilian Nursing, shown in the study of the Brazilian Nursing profile carried out by the Federal Nursing Council (*Conselho Federal de Enfermagem*, COFEN)^(14)^. A sociodemographic analysis was also carried out in this study, evidencing the gaps that might be improved and where greater efforts should be concentrated, especially in a profession that has at its core the qualification and instrumentalization of managerial processes, which lead to improvement in care aspects. In delivering their competencies, nurses equate basic constructs of the profession that guide and encourage their actions^(15)^.

These professionals seek to qualify their knowledge in the pursuit of professional improvement, both through short-term technical courses and through courses with longer hour loads, such as *lato sensu* specializations, where most of the nurses who answered the instruments stated that they were specialized in some specific Nursing area. In this sense, a very positive movement can be seen focused on adding value to the professional profile and technical competence to the curriculum, with professional development as a guideline for actions. In identifying the courses where these professionals specialized the most, for more than 50%, the specialization in urgency and emergency was highlighted; in other words, nurses chose to specialize in the area where they perform their care functions, which certainly qualifies the assistance provided and, therefore, care provision, the final assistance product.

Another aspect worth noting is the fact that the professionals attend training/improvement courses in the urgency and emergency area, such as BLS, ACLS, ATCN and PHTLS, among others, which shows interest in improving the technique to act more effectively in the urgency and emergency sector, where these skills are required, due to its dynamism and complexity. Linked to this, rapid and assertive reasoning demands training, qualification and hours of dedication and studies. The professionals are committed to seeking qualifications that underlie their daily practice and exercise, which are also forms of support and personal commitment to professional growth, further raising the assumptions of the profession^(16)^. A similar movement was identified in a study where the nurses identified the need for training through self-perception^(17)^.

A study evidenced the need for standardization of the training opportunities in the urgency and emergency sectors^(18)^, precisely because for understanding that the actions carried out by those who are submerged in the art of care go beyond prescriptive approaches, as they deepen and ground knowledge. Qualifying the assistance provided means being completely devoted to care and finding ways and possibilities to contribute to it, also through reflective thinking, which seeks new, technology-driven ways that serve research and development^(19-20)^. This is a construction that should be sharpened in nurses, as their contribution to the growth and development of society is paramount. Their academic background already points to this factor, pointing out the social dimension of the nursing profession^(21)^.

When comparing self-assessment and hetero-assessment, in most factors, nurses self-assessed better than their managers, however, in some factors, the assessment made by the manager (hetero-assessment) was higher than that performed by the nurse. This result was different from the one found in the ECAEE validation study^(6)^. It is worth noting that the instruments were filled out during working hours and that, even due to the dynamics of the urgency and emergency sector, some items may have gone unnoticed.

In the comparison between the self- and hetero-evaluation scores, the factors with the highest scores were as follows: in the self-evaluation, Professional practice and Targeted action and in the hetero-evaluation, Professional excellence. In fact, nurses’ professional competencies are supported by actions that tend to qualify them^(22)^, as these professionals, through all the investment they make in their career, do not seek anything more than materializing these competences. They face these challenges because they know that, in order to carry out an emancipating and rehabilitating professional practice for the patients, it is necessary to surround themselves with theoretical and practical knowledge with a view to professional excellence^(23)^. A similar movement was found in a study carried out in Chinese military hospitals, where the need for professional qualification based on nurses’ experiences and expectations was evidenced^(24)^.

Realizing this coherence of answers between clinical nurses and managers is a positive aspect, as it shows concern between the parties, which means that those who care are having their actions observed and those who observe are guiding their managerial perspective by instruments validated in the scientific literature, that is, Nursing using instruments validated in the category studies to assess the group itself.

Relationships at work confront the knowledge of Being a nurse, as they need to manage their team of nursing technicians and have a good experience with the multiprofessional team, without losing their professional identity, being authentic in the name of the care they elaborate, articulate and provide to the patients^(25)^. In this sense, the challenge becomes positive and, at the same time, provocative, as nurses direct their actions towards care, which will be crowned with professional excellence, which confers even more authenticity and strengthening to identification of the category.

The APROCENF scale^(5)^ also obtained a significant number of assessments. Each nurse answered this instrument fifteen times as a way of portraying their daily routine in the urgency and emergency sector as accurately as possible. It is noteworthy that this scale was evaluated in all sectors of the urgency and emergency unit, reflecting assistance from the emergency room to medication.

Although it had not yet been tested in urgency and emergency units, a “Good” APROCENF score was obtained - such response was reflected in the eight domains of the scale - which means that the final product of the nurses’ actions required towards the patients in this study was quite expressive. Obviously, an “Excellent” score in the APROCENF scale would be the most appropriate; however, it is understood that this indication is already a starting point for the managers of these units to work with the permanent education sector on these issues, with the purpose of training professionals to deliver a care product based on even more solid competencies and be encouraged to develop those they do not yet have. In this sense, this study does meet the scope, when comparing its results to another research in which the APROCENF scale was applied in specialized units^(26)^, where the result of the care product delivered by Nursing was also predominantly “Good”.

In the correlation between the mean values of the fifteen evaluations of the APROCENF scale carried out by the nurses and the professional competences measured by ECAEE, significant differences were observed both in the self- and in the hetero-evaluation, which shows that specific competencies are required for planning, implementation and delivery of the product care in the urgency and emergency unit. To a greater or lesser extent, all ECAEE factors were related to the domains from the APROCENF scale in the nurses’ assessments, as well as in their managers’.

Factors such as Professional practice^(6)^, Relationships at work, Positive challenge, Targeted action, Constructive behavior and Adaptation to change were directly related to Nursing staffing, as they enable processes that require specific human resources to act directly in the health-disease process, placing their professional competencies in favor of an effective rehabilitation process that interacts dynamically in restoring the patients’ health, with the aim of reintegrating them into family and social life.

Staffing is one of the impacting factors in Nursing care, as an adequate number of professionals for the expressive care demand exerts a direct effect on the patients’ rehabilitation process. Unfortunately, in many urgency and emergency scenarios, which already have the aggravating factor of overcrowding, operating in an open-door system with spontaneous demand, the staff is reduced, causing overload, leaves and absenteeism. On the other hand, a study showed that an adequate number of nurses in patient care reduced mortality, readmissions and the patients’ hospitalization times^(27)^.

Patient satisfaction in a hospital unit involves a set of actions, reflecting the care provided by nurses as well as the implementation and execution of care actions by their team. Nurses provide quality care when the conditions of the service are favorable for them to carry out their routine, and this makes the care processes possible, developing a chain reaction where the professionals’ satisfaction has its direct reflection in the care provided to the patients, consecutively resulting in improvements in their rehabilitation process^(28)^.

In the aforementioned study^(28)^, the fact of having good Nursing staffing met the demand of the patients participating in the research. With this assumption as a starting point, we conceive that the APROCENF scale^(5)^ would possibly have a more expressive evaluation, showing excellence in the quality of service provision and delivery of the most effective care product, as long as there are enough nurses in urgencies and emergencies.

Likewise, care monitoring and transfer^(5)^ were related to the professional practice of nurses who, through their expertise, develop good relationships with the team as a whole, training them to adapt to change, while being flexible to it, managing conflicts that may arise towards a larger process, which is a positive challenge.

Care must be continuous, so that the processes initiated in the unit where the patients are located are feasible, with a sequence in all the in-hospital sectors to which they are transferred, even in the post-discharge period, where articulation with the network services should provide this follow-up, avoiding readmissions^(29)^.

Care non-continuity increases the patients’ hospitalization time and, if the patients are already at their home, it contributes to readmissions, which requires professionals who perform this transfer of essential competencies to the service they operate^(30)^. In this sense, the nurses’ competencies in urgencies and emergencies confer solidity to this process, through development of practices for care continuity, optimizing in-hospital transfers as well as home care, with communication as a factor and a predominant competence. A study pointed out that communication is one of the factors that interfere with care transfer^(31)^ since, at the same time that it enables and leverages the care process, its absence can impair this process.

Failures in this process imply an increase in hospitalization costs, stress for patients and their families and blockage in bed turnover^(31)^. Regarding care transfer in relation to the APROCENF scale^(5)^, a “Good” level was obtained in the score, which implies a closer analysis by the managers who, along with the permanent education sector, should create conditions that optimize this process, so that patient care is not impaired and their transfers and discharges are not postponed.

It is noticed that all the highlighted competencies converge to meet the care needs, with a view to promoting the health and well-being of the patients, who find support for their real health needs in Nursing care^(5)^. Prioritizing care focused on the patients’ real health needs, through targeted care planning, will meet their health demands^(32)^ and, with that, nurses reassert their competencies in the perspective of developing many others that find shelter in the patients in the scope of the needs that they bring to the service in search of resolution.

The “Nursing staffing” and “Care transfer” domains were related to several competencies, pointing out that, in order to provide effective assistance, an adequate number of professionals is necessary, so that they can transfer care in the safest possible way, which implies coordination between Nursing management and the unit’s permanent education service to make this process viable.

The limitation of this study is due to the fact that the instruments are answered without fail during the shifts, which is why, considering the dynamics of the urgency and emergency unit, some items may not have reflected exactly what the nurses wanted to express. However, it was found that the APROCENF scale can be used in emergency units, especially in association with ECAEE, where, in addition to confronting the competencies they have, the professionals will strive to develop those that are limiting factors in the assistance they provide, precluding better delivery of the Nursing care product.

## Conclusion

The nurses’ competencies are related to delivery of the Nursing care product in the urgency and emergency context. Such competencies were identified in the seven factors: however, there was greater emphasis on “Professional practice”, “Relationships at work”, “Positive challenge”, “Targeted action”, “Constructive behavior” and “Professional excellence” both in the in self- and in the hetero-evaluation. While identifying the competencies, the nurses carried out their self-evaluation based on the specificity of the urgency and emergency service, taking into account the complexity of the sector and grounding their critical perspective on unusual events that arrive at the unit and require specific competencies for an effective and efficient approach.

The APROCENF scale identified the Nursing care product as “Good”, showing that delivery of Nursing care is well evaluated by those who are providing this service.

Given the above, we found that there is a relationship between professional competencies distributed in the ECAEE factors through the dimensions of the APROCENF scale, highlighting “Nursing staffing”, “Care monitoring and transfer” and “Meeting the care needs” which, when directly interfering in the assistance that generates a product to be consumed by the patients, characterizing the profile of the nurses who guide their care actions through the competencies listed in this study.

## References

[1] Soares MI, Leal LA, Resck ZMR, Terra FS, Chaves LDP, Henriques SH (2019). Competence-based performance evaluation in hospital nurses. Rev. Latino-Am. Enfermagem.

[2] Moreira DA, Ferraz CMLC, Costa IP, Amaral JM, Lima TT, Brito MJM (2020). Professional practice of nurses and influences on moral sensitivity. Rev Gaúcha Enferm.

[3] Silva LAS, Dias AK, Gonçalves JG, Pereira NR, Pereira RA (2019). Nursing and emergency action in nursing. Rev Extensão [Internet].

[4] Alves M, Melo CL (2019). Handoff of care in the perspective of the nursing professionals of an emergency unit. Rev Min Enferm.

[5] Cucolo DF, Perroca MG (2017). Assessment of the nursing care product (APROCENF): a reliability and construct validity study. Rev. Latino-Am. Enfermagem [Internet].

[6] Holanda FL, Marra CC, Cunha ICKO (2019). Professional competence of nurses in emergency services: evidence of content validity. Rev Bras Enferm.

[7] Bukoh M, Siah C (2020). A systematic review on the structured handover interventions between nurses in improving patient safety outcomes. J Nurs Manag.

[8] Jesus JA, Balsanelli AP (2020). Competências do enfermeiro em emergência e o produto do cuidar em enfermagem: revisão integrativa. Rev Rene.

[9] Polit DF, Beck CT (2018). Fundamentos de pesquisa em enfermagem: avaliação de evidências para a prática de enfermagem.

[10] STROBE (2022). Strengthening the reporting of observational studies in epidemiology. STROBE Checklists [Internet].

[11] Gottems LBD, Carvalho EMP, Guilhem D, Pires MRGM (2018). Boas práticas no parto normal: análise da confiabilidade de um instrumento pelo Alfa de Cronbach. Rev. Latino-Am. Enfermagem [Internet].

[12] Machado MH (2017). Perfil da enfermagem no Brasil: relatório final [Internet]. http://www.cofen.gov.br/perfilenfermagem/pdfs/relatoriofinal.pdf.

[13] Ribeiro OMPL, Martins MMFPS, Tronchin DMR, Silva JMAV, Forte ECN (2019). Professional practice models used by nurses in Portuguese hospitals. Rev Bras Enferm.

[14] Sanjuan-Quiles A, Hernández-Ramón MP, Juliá-Sanchis R, García-Aracil N, Encina MEC, Perpiñá-Galvañ J (2019). Handover of patients from prehospital emergency services to emergency departments: A qualitative analysis based on experiences of nurses. J Nurs Care Qual.

[15] Batista REA, Peduzzi M (2019). Interprofessional Practice in the Emergency Service: specific and shared assignments of nurses. Rev Bras Enferm.

[16] Campanha RT, Magalhães AMM, Oliveira JLC, Kreling A, Riboldi CO (2020). Leadership in brazilian hospital nursing: contributions to the quality of patient care and safety. Res Soc Dev [Internet].

[17] Ndung’u A, Ndirangu E, Sarki A, Isiaho L (2022). A Cross-sectional Study of Self-Perceived Educational Needs of Emergency Nurses in Two Tertiary Hospitals in Nairobi, Kenya. J Emerg Nurs.

[18] Calder S, Tomczyk B, Cussen ME, Hansen GJ, Hansen TJ, Jensen J (2022). A Framework for Standardizing Emergency Nursing Education and Training Across a Regional Health Care System: Programming, Planning, and Development via International Collaboration. J Emerg Nurs.

[19] Olino L, Gonçalves AC, Strada JKR, Vieira LB, Machado MLP, Molina KL (2019). Effective communication for patient safety: transfer note and Modified Early Warning Score. Rev Gaúcha Enferm.

[20] Holanda F, Marra CC, Meireles ECA, Balsanelli AP, Cunha ICKO (2022). Lilalva Scale: soft-hard technology to measure clinical competencies in emergencies of nurses. Rev Bras Enferm.

[21] Silva AGI, Silva FJN, Costa F, Alcântara GC, Costa GF (2021). Boas práticas de liderança do enfermeiro no contexto hospitalar. Rev Nurs.

[22] Chotolli MR, Cucolo DF, Perroca MG (2018). Assessment of the product of nursing care in specialized hospitals. Rev Bras Enferm.

[23] McHugh MD, Aiken LH, Sloane DM, Windsor C, Douglas C, Yates P (2021). Effects of nurse-to-patient ratio legislation on nurse staffing and patient mortality, readmissions, and length of stay: a prospective study in a panel of hospitals. Lancet.

[24] Huijuan M, Suofei Z, Xiaoli Z, Jinyu H, Zhen C, Yu L (2023). Continuing professional education experiences and expectations of nurses in Chinese military hospitals: A quantitative and qualitative study. Nurse Educ Today.

[25] Mello TS, Miorin JD, Camponogara S, Paula CC, Pinno C, Freitas EO (2021). Factors that influence for effective intra-hospital care transfer: Integrative review. Res Soc Dev.

[26] Desmedt M, Ulenaers D, Grosemans J, Hellings J, Bergs J (2021). Clinical handover and handoff in healthcare: A systematic review of systematic reviews. Int J Qual Health Care.

[27] Lavoie P, Clausen C, Purden M, Emed J, Frunchak V, Clarke SP (2021). Nurses’ experience of handoffs on four Canadian medical and surgical units: A shared accountability for knowing and safeguarding the patient. J Adv Nurs.

[28] Methangkool E, Tollinche L, Sparling J, Agarwala AV (2019). Communication: is there a standard handover technique to transfer patient care?. Int Anesthesiol Clinics.

[29] Hemesath MP, Kovalski AV, Echer IC, Lucena AF, Rosa NG (2019). Effective communication on temporary transfers of inpatient care. Rev Gaúcha Enferm.

[30] Tortosa-Alted R, Reverté-Villarroya S, Martínez-Segura E, López-Pablo C, Berenguer-Poblet M (2021). Emergency handover of critical patients. A systematic review. Int Emerg Nurs.

[31] Moraes KB, Riboldi CO, Silva KS, Maschio J, Stefani LPC, Tavares JP (2019). Transfer of the care of patients with low risk of mortality in postoperative: experience report. Rev Gaúcha Enferm.

[32] Létourneau D, Goudreau J, Cara C (2021). Nursing Students and Nurses’ Recommendations Aiming at Improving the Development of the Humanistic Caring Competency. Can J Nurs Res.

